# The Effect of Curcumin on the Prevention of Myringosclerosis in Rats

**DOI:** 10.4274/tao.2020.5827

**Published:** 2021-03-26

**Authors:** Özlem Akkoca, Selda Kargın Kaytez, Nihat Yumuşak, Akif Sinan Bilgen, Ali Kavuzlu, Ramazan Öcal, Hatice Çelik, Necmi Arslan

**Affiliations:** 1 Department of Otorhinolaryngology, University of Health Sciences Ankara Training and Research Hospital, Ankara, Turkey; 2 Department of Pathology, Harran University Faculty of Veterinary Medicine, Şanlıurfa, Turkey

**Keywords:** Myringosclerosis, curcumin, tympanic membrane, rats, histology, microscopy, animal experiment

## Abstract

**Objective:**

The aim of this study was to investigate the preventative effect of oral curcumin (CMN) on myringosclerosis (MS) in an experimental rat model.

**Methods:**

The study included 21 female Wistar albino rats randomly separated into three groups. Group 1 was given no treatment (control group). In Group 2 and Group 3, the tympanic membrane (TM) was perforated using a sterile ear pick. The rats in Group 3 were administered oral CMN 200 mg/kg/day. All rats were sacrificed after 16 days. Otomicroscopic and histopathologic examinations were performed on the tympanic membranes.

**Results:**

Histopathologic examinations revealed that there were statistically significant differences between Group 2 and Group 3 in terms of MS degrees (p<0.001) and mean thicknesses of TMs (p<0.001), but there were no differences between Group 1 and Group 3. In respect of MS detected by otomicroscopy, a statistically significant difference was determined between Groups 1 and 2 (p<0.001) and between Groups 2 and 3 (p<0.01), but there was no significant difference between Group 1 and Group 3 (p=0.575).

**Conclusion:**

Orally administered CMN can prevent myringosclerosis formation in experimentally induced myringotomies.

## Introduction

Myringosclerosis (MS) is an irreversible degeneration characterized by dystrophic calcification and hyalinization in the fibrous layer of the tympanic membrane (TM) (1). It is caused by middle ear infections or surgical intervention to the TM (2). Especially in the treatment of otitis media with effusion in children, the incidence of MS has been reported as 28%-61% depending on the frequency of ventilation tube application (3, 4). Recent studies have shown that hyperoxidative damage and free oxygen radicals may be the main cause of MS formation (1, 5). It is therefore thought that with the application of anti-inflammatory or antioxidant agents, which can prevent the harmful effects of oxygen-derived free radicals, it may be possible to reduce or prevent the development of MS (1, 4).

Curcumin (CMN) (diferuloylmethane) is a natural polyphenol product obtained from the rhizomes of the Curcuma longa plant (6). In recent years, it has been reported that CMN, which has been used in medical preparations and food coloring for centuries, has strong antioxidant and anti-inflammatory effects (6, 7). It is a safe, non-toxic, and well tolerated agent even at high doses (8). There are studies showing that intraperitoneal, oral, and topical CMN application has a positive effect on wound healing in different parts of the body (8, 9). There are, however, no studies that have investigated the effect of oral CMN administration for the prevention of MS development.

The aim of this study was to investigate whether CMN has a beneficial effect in preventing MS development. To evaluate the effectiveness of CMN, MS was experimentally induced in rats and the results were presented with histopathological parameters.

## Methods

### Approval and Animals

Approval for this experimental study was obtained from the Experimental Animal Research Ethics Committee of University of Health Sciences, Ankara Training and Research Hospital (Approval Date: September 17, 2019; Approval Number: 596). The procedures required for the care and treatment of all animals were carried out in accordance with the ethical principles of animal experiments and the laws related to animal protection. The animals were housed in standard cages under a 12-hour light/dark cycle, at a stable temperature of 20°±2 °C and humidity of 60%±5%, with food and water ad libitum.

### Curcumin Preparation

CMN was administered orally via a feeding tube as 200 mL/kg/day (Sigma-Aldrich Chemical Co.; St. Louis, MO, USA) diluted with 1 mL saline. CMN doses were chosen based on previous experimental studies (10, 11, 12).

### Experimental Design and Surgical Procedure

A total of 21 female Wistar albino rats, aged 10-12 weeks, each weighing 250 g, were used in the study. All rats were anesthetized with ketamine hydrochloride (50 mg/kg, intramuscular) (New York, NY, USA) preoperatively, then examined with an otomicroscope (Opmi 1; Zeiss, Germany). Only rats with normal TMs examination were included in the study. Rats with TM perforation, myringosclerosis, and infection in the external auditory canal and/or TM were excluded from the study. Myringotomy was performed bilaterally, approximately 2 mm in the upper posterior quadrant of the TMs, by the same researcher, under the otomicroscope, using a sterile ear pick and ear speculum. No signs of ear infection were detected in any animal during the study.

The rats were randomly separated into three groups of 7 (Groups 1, 2, 3). Myringotomy was performed in Group 2 and Group 3 using a sterile ear pick. The groups were treated as follows:

Group 1 (control group): normal TM was confirmed, and no treatment was applied,

Group 2: myringotomy was performed,

Group 3: myringotomy was performed followed by oral CMN solution 200 mg/kg/day administered with a feeding tube for 15 days as treatment.

Only one rat in Group 1 died during this experiment and was excluded from the study.

### Otomicroscopic Examination

On the 16th day of the study, the TMs of 20 rats (40 ears) were evaluated by otomicroscopy under anesthesia with ketamine, and then the rats were sacrificed. Myringosclerotic lesions were scored semi-quantitatively using a 4-point scale; 0: no visible myringosclerotic plaques, 1: MS that can be seen at certain intervals with white halo around umbo, 2: Moderate MS containing white halo around umbo, and visible along the manubrium mallei and the anulus, 3: Severe MS with horseshoe-like whitish deposits along the anulus.

### Histopathological Examination

After otomicroscopic evaluation, the rats were sacrificed with a high dose of pentobarbital (80 mg/kg, intraperitoneal injection). After decapitation, the head and jaw muscles of the rats were cleaned. Then, the middle ear was removed with the help of bone scissors with the bulla. The tissues taken were decalcified in EDTA solution for 7 days. Then TMs were separated under the stereomicroscope and placed in neutral formal with the bulla and fixed for histopathological analysis. Histopathological evaluation was performed under a light microscope by a pathologist blinded to the groups. All tissues were fixed overnight with a 10% buffered neutralized formalin solution, followed by decalcification with a 10% nitric oxide solution. Routine pathology procedures were applied, and the samples were embedded in paraffin blocks after passing through graded alcohol (50%, 75%, 96%, 100%) and xylol series. Sections were taken from the parts of the TM where myringotomy was performed. Sections of 5 µ thickness obtained from the prepared blocks were taken on the slides of a Leica RM 2125 RT including the first three sections and every tenth section. The preparations were stained with hematoxylin-eosin (H&E) after treatment with alcohol and xylol. Sclerotic changes in the connective tissue of the lamina propria were evaluated with Masson Trichrome staining. All samples were examined under a high-resolution light microscope (Olympus DP-73 camera, Olympus BX53-DIC microscope; Tokyo, Japan).

Sclerotic changes and density of fibroblast proliferation (FP) in the TM lamina propria were separately quantitatively rated; 0: no visible myringosclerosis/FP, 1: mild myringosclerosis/FP, 2: moderate myringosclerosis/FP, 3: severe myringosclerosis/FP. TM thickness (TMT) was measured using the H&E stained sections of the preparations. The images obtained under the microscope were digitalized with a camera (Olympus BX50, Olympus Optical Co., Tokyo, Japan) and transferred to a computer environment. The TMTs in 10 different areas were measured in micrometers. For statistical analysis, the mean of these 10 values was accepted as the TMT value.

### Statistical Analysis

Statistical analyses of the data obtained in the study were made using the IBM Statistical Package for the Social Sciences v20 (IBM SPSS Corp.; Armonk, NY, USA) software. Descriptive statistics were stated as mean±standard deviation (SD), median, minimum, and maximum values for continuous data. Kruskal-Wallis Variance Analysis was used in the analysis of the differences of the variables in three groups. The group/groups from where the difference originated was/were determined using the Kruskal-Wallis Multiple Comparison test. A value of p<0.05 was accepted as statistically significant.

## Results

In the otomicroscopic examination on the 16th day all the TM perforations were observed to have healed in all three groups. The myringosclerotic lesions were evaluated in 4 grades. In the histopathological examination, sclerotic changes, FP, and mean TMT were determined (Table 1, Table 2).

### Otomicroscopic Examination

In Group 2, myringosclerosis was detected in 12 ears (85.7%) as grade 3, and in two ears (14.3%) as grade 1, and in Group 3 in four ears (28.6%) as grade 1, in two ears (14.3 %) as grade 2 and in eight ears there were no sclerotic changes (57,1%). No sclerotic changes were detected in Group 1 (Table 1). In respect of the myringosclerosis groups detected with otomicroscopy, the differences between Groups 1 and 2 (p<0.001), and between Groups 2 and 3 (p<0.01) were determined to be statistically significant. There was no significant difference between Groups 1 and 3 (p=0.575).

### Histopathological Examination

**Figure 1:**
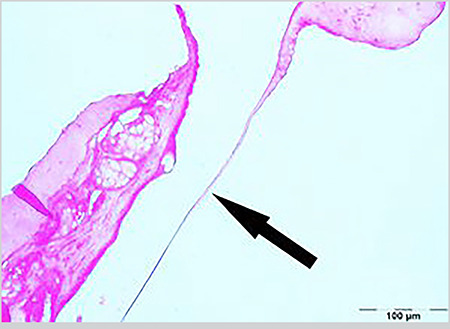
View of normal tympanic membrane (0 point scale) in (hematoxylin and eosin, original magnification x200)

**Figure 2:**
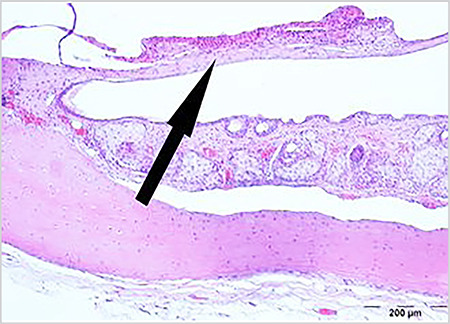
Sclerotic areas and thickness in the tympanic membrane from Group 2

**Figure 3:**
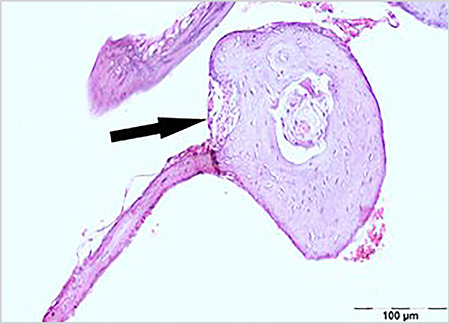
Sclerotic areas and thickness in the tympanic membrane from Group 3

Sclerotic changes in the TM lamina propria were shown in Table 2 and Figures 1, 2, 3. A statistically significant difference was found between the groups in terms of sclerotic changes in the lamina propria detected in histopathological examination (p<0.001). Statistically significant difference was determined between Groups 1 and 2 (p<0.001), and between Groups 2 and 3 (p<0.01). The difference between Groups 1 and 3 was not statistically significant (p=0.538).

FP in lamina propria of TM was detected as follows: in Group 2 severe (71.4%) in 10 ears, moderate (14.3%) in two ears, and mild (14.3%) in two ears; in Group 3 mild in eight ears (57.1%), and in Group 1 mild in two ears (16.7%). Statistically significant difference in FP in the lamina propria was found between Groups 1 and 2 (p<0.001), and between Groups 2 and 3 (p<0.01). There was no significant difference between Groups 1 and 3 (p=0.176).

The average TMT was determined in Group 1 as 12.5 µm (range, 9-38), in Group 2 as 178.5 µm (range, 13-196), and in Group 3 as 41 µm (range, 29-131) (Table 3) (Figures 1, 2, 3). Statistically significant difference was determined between Groups 1 and 2 (p<0.001), between Groups 2 and 3 (p<0.05), and between Groups 1 and 3 (p<0.01) (Table 3).

## Discussion

Based on otomicroscopic and histopathological findings, the results of this study demonstrated that oral administration of CMN, a nutrient known to have antioxidant and anti-inflammatory effects, prevents the development of experimentally induced MS.

MS is a non-specific, irreversible chronic inflammatory condition characterized by calcium deposition and hyalinization in the lamina propria layer of the TM, typically presenting as white sclerotic lesions (4, 5). Although MS is observed especially in children due to myringotomy and ventilation tube applications, factors such as chronic middle ear infections and trauma have also been held responsible in the etiology (5). Mattsson et al. (13) reported that MS had developed in nine hours after myringotomy in pars tensa and in 12 hours in pars flaccida. In otomicroscopic examination, lesions seen as white calcified plaques of variable shapes and sizes in the TM have been observed in histopathological examination to be mineralized aggregates with calcium phosphate content located in irregular collagen fibers in the lamina propria of sclerotic lesions (14, 15). Following acute TM perforation, TM thickness increases due to edema, inflammation, and new vessel formation in the fibrous layer. It also shows increased fibroblastic activity and epithelial proliferation in TM (16).

Although the formation mechanism is not fully known, recent studies have shown that a hyperoxic environment formed in the middle ear as a result of TM trauma and perforations, and subsequently, free oxygen radicals (ROS) appear (1, 2, 4, 5).

In humans and animals, the middle ear cavity contains 5.5-12.1% oxygen (17). After myringotomy, atmospheric air enters the middle ear cavity and the O2 level increases in the middle ear. Then ROS formation increases in the mitochondria and the endoplasmic reticulum (18). Polymorphonuclear cells and macrophages migrate to the inflammation site, leading to phagocytosis and increased arachidonic acid metabolism. Thus, increasing amounts of ROS contribute to the development of MS (16).

The ROS formed lead to the development of MS by initiating the inflammatory process and tissue damage, creating irregular collagen synthesis, hyaline degeneration, and calcification (19). ROS can be neutralized by substances with antioxidant properties. There are many studies which show that MS formation can be prevented by applying antioxidant, anti-inflammatory, and ROS scavenging agents (1, 2, 3, 4, 16, 20, 21, 22).

Eğilmez et al. (1) reported that oral or topical administration of Hypericum perforatum extract after myringotomy decreased the inflammation and fibroblastic activity in the lamina propria of the TMs of the rats. Dündar et al. (2) demonstrated that ascorbic acid and/or N-acetylcysteine treatments reduced the development of MS by reducing inflammation scores and cellular infiltration. Üstündağ et al. (3) found that topical application of dexamethasone in rats after myringotomy has positive effects in reducing both the severity and prevalence of MS. Kargin Kaytez et al. (4) used montelukast orally and topically in rats after myringotomy and reported that it reduced FP and TM thickness. Kazikdas et al. (20) suggested that the prevalence of myringosclerotic plaques was less in rats receiving alpha-tocopherol intramuscularly. Park et al. (21) proved that sodium thiosulphate reduced the tympanic membrane thickness after myringotomy in rats and reduced the formation of MS by preventing calcium accumulation. Görür et al. (22) observed in a study that intraperitoneal selenium administration during the closure period of the perforation following myringotomy in rats reduced the occurrence of MS. Vuralkan et al. (16) concluded that FP and TM thickness decreased with both local and intraperitoneal application of L-carnitine. However, there are no studies showing the effect of oral CMN administration on MS development. Our study is the first study in the literature, demonstrating that oral CMN administration after myringotomy in rats decreases both FP and TM thickness in the lamina propria.

CMN is a type of spice that is commonly used in South Asian cuisines. In recent years it has been shown to have anti-inflammatory, antioxidant, wound-healing, hypoglycemic and antimicrobial properties. It is used to prevent and treat diseases related to the digestive system, the cardiovascular system, the liver, and the skin, as well as those of the endocrine system such as diabetes, and has positive effects on wound healing (8, 9). In rat studies, it has been found that orally administered CMN increases the activity of antioxidant enzymes such as superoxide dismutase, catalase, and glutathione peroxidase (23). These antioxidant enzymes have a protective effect on human cells against the harmful effects of toxic reactive oxygen radicals. It has also been shown that CMN inhibits the production of proinflammatory cytokines released from monocytes and macrophages, such as tumor necrosis factor alpha and interleukin-1, which are known to play important role in regulating inflammatory responses (24). In the early phase of wound healing, CMN also increases the speed of healing by inducing apoptosis of inflammatory cells and shortens the inflammatory process. CMN has an enhancing effect on collagen synthesis and has an effect on the differentiation of fibroblasts and facilitating migration to the wound site (25).

There are many studies which show that CMN, known to have significant antioxidant and anti-inflammatory effects, can be used as a wound-healing agent, especially when administered topically (9, 25, 26). However, there are few studies that discuss its oral administration (10, 27). In a literature review, a study was found which evaluated the improvement in TM after paracentesis using topical CMN, but no other study could be found that examined the effect of CMN on oral administration and MS formation. In a study by Birdane et al. (26) topical CMN was used in the form of drops and it was found that TMs healed close to normal in rats after paracentesis similar to normal TMs. In the same study, it was reported that TMT and sclerosis level were significantly lower in the CMN group compared to the control group. In our study, CMN was administered orally 200 mg/kg/day diluted with 1ml saline via a feeding tube to the rats in Group 3 for 15 days as has been described in previous studies (10, 11, 12). Correlations were determined between otomicroscopic and histopathologic examination results. In both examinations, statistically significant difference was found between Groups 1 and 2 (p<0.01), and between Groups 2 and 3 (p<0.01), but there was no significant difference between Groups 1 and 3 (p=0.575, p=0.538) (Tables 1, 2). However, when comparing the numbers of ears detected with sclerosis and the grades in both examinations (Tables 1 and 2), it was seen that mild sclerosis could be detected in the histopathologic examination but not in the otomicroscopic examination. In Group 3, histopathologic examination found moderate sclerosis in four ears (28.6%) and mild sclerosis in six ears (42.9%), while otomicroscopic examination found MS grade 1 in four ears (28.6%) and grade 2 in two ears (14.3%). Although otomicroscopy has 80% sensitivity and 75% specificity, as stated by Santos et al. (28), it can also be seen from the findings of our study that histolopathological examination is a better method to evaluate MS. Sclerosis in the lamina propria and FP in rats that were administered oral CMN had similar levels of TM characteristics as the rats in the control group, and only the mean TMT was higher (Figures 1, 2). The mean TMT value was found to be highest at 178.5 µm (13-196) in Group 2, and lowest at 12.5 µm (9-38) in Group 1. Although no difference in sclerosis and FP was determined between Group 1 (control group) and Group 3 (oral CMN group), TMT was at a higher level in Group 3, which may have resulted from the effect of CMN enhancing myofibroblasts, fibroblasts, and macrophages migration and angiogenesis in the post-traumatic healing process (29).

In this study, we found that orally administered CMN can prevent the occurrence of experimental MS. Therefore, CMN, which is known as an important nutrient, should be part of the daily diet, and topical use should be considered in those with infection caused by a ventilation tube or myringotomy. There is a need for further clinical studies in this field.

## Conclusion

Oral administration of CMN, which is known and used as a spice, can prevent experimental MS. These effects of CMN may be due to the antioxidant and/or anti-inflammatory properties. However, there is a need for clinical trials which can reveal the effects of CMN on MS.

**Main Points**

• Oral curcumin has an inhibitory effect on sclerotic changes and fibroblast proliferation in the lamina propria layer of the tympanic membrane in experimental myringosclerosis.

• Oral intake of curcumin in experimental myringosclerosis has a decreasing effect on tympanic membrane thickness.

• Curcumin can prevent experimental myringosclerosis, due to its antioxidant and/or anti-inflammatory effects when administered orally.

## Figures and Tables

**Table 1:**
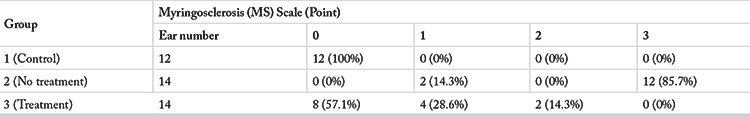
Otomicroscopic examination of myringosclerosis

**Table 2:**
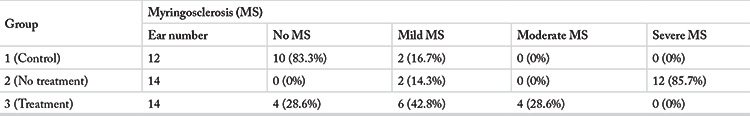
Histopathologic examination of sclerosis

**Table 3:**
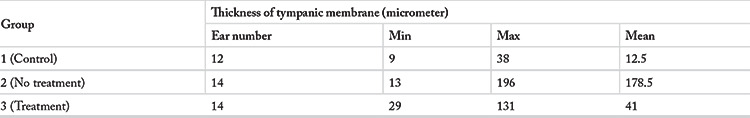
Thickness of tympanic membrane
